# Probing transcription factor subsets in gene regulatory networks

**DOI:** 10.1186/s13015-026-00297-x

**Published:** 2026-05-15

**Authors:** Lukas Geis, Dennis Hecker, Martin Hoefer, Ulrich Meyer, Marcel H. Schulz

**Affiliations:** 1 Institute of Computer Science, Goethe University Frankfurt, Robert-Mayer-Straße 11-15, Frankfurt am Main, 60325 Hesse Germany; 2 Institute for Computational Genomic Medicine, Goethe University Frankfurt, Theodor-Stern-Kai 7, Frankfurt am Main, 60590 Hesse Germany; 3 Department of Computer Science, RWTH Aachen, Ahornstraße 55, Aachen, 52074 NRW Germany

**Keywords:** Probing problem, Maximum coverage problem, Transcription factors, TF-gene networks

## Abstract

Transcription factors (TFs) are essential players in the regulation of gene expression and thus have been the subject of interest in the context of diseases and the manipulation of specific cell functions or pathways. The complex interplay of TFs and genes can be modeled as a network, in which each edge represents a regulatory influence. A challenge in such networks is the identification of a set of TFs with the maximum regulatory influence, which is of particular importance in the context of perturbation studies. We model the task of finding a set of TFs to maximize the influence on a set of genes as a probing problem in a bipartite graph. We test different adaptive and non-adaptive algorithms on simulated graphs and discuss their properties and adaptivity gap. Then, we apply the algorithms on real-life data to find TFs regulating genes involved in T-cell mediated immunity and lymphoid leukemia. The approach has minimal data requirements and can be readily applied to all other types of bipartite networks.

## Introduction

Transcription factors (TFs) are regulatory proteins that influence the expression of genes by binding to the DNA in a sequence-specific fashion. TFs specifically bind accessible regions of the chromatin, which are also referred to as regulatory elements (REs) [1–3]. Due to their central role in numerous biological processes and their involvement in diseases, TFs are the subject of ongoing research. To understand the function of a TF, it is essential to know its target genes. There are various approaches to map TFs to genes, such as relying on databases that collect TF-gene interactions [4–7], correlating expression of genes and TFs across a collection of samples or single cells [8, 9], comparing changes between conditions [10, 11], observing the consequences of the perturbation of TFs [12–15], or by annotating transcription factor binding sites (TFBS) originating either from experimental data, such as ChIP-seq [16, 17], or from predictions based on known sequence binding preferences [2]. Since a gene is not only influenced by TFs binding in its immediate vicinity, all REs of a gene should be considered as locations with potential TFBS [3]. The relation between genes and TFs is often modeled via networks, in which each edge represents a regulatory influence [9, 18–21].

A challenge in such networks is the identification of TFs that regulate a gene set of interest, for example, genes of a specific pathway or disease. Commonly, the importance of TFs is assessed one at a time [22–25]. However, TFs are known to act cooperatively and pathways can be regulated by a set of TFs [1–3, 26]. A famous instance of this is the four Yamanaka factors Oct3/4, Sox2, c-Myc, and Klf4 [27]. The identification of a set of TFs in a network, either with the goal of formulating hypotheses about how a pathway is regulated or for finding potential targets for perturbation experiments, can become a combinatorial problem. For example, picking three TFs out of 400 TFs results in over 10 million possible combinations. Few works exist that explicitly aim to find sets of TFs. Gross and Blüthgen [28] intended to identify combinations of perturbations that maximize the gained knowledge about the relationships within a biological network by describing it as a maximum-flow problem. Similarly, the tool MEED suggests perturbation experiments that maximize the knowledge about regulatory relationships with a predictive logical model [29]. Wang et al. [30] built TF regulation models per gene based on perturbation data sets, which they subsequently used to define sets of perturbations which would be most informative for understanding the gene’s regulation. While these approaches promise to help in designing experiments to validate the network, they do not aim to find sets of TFs with the maximum coverage of genes in the network.

We address the task of finding a set of TFs to maximize the number of target genes, i.e. the genes that are regulated by the TFs, by solving a stochastic probing problem. The probing problem asks to select a subset of elements of a given ground set that maximizes a given stochastic objective function [31–33]. Formally, for a given set $A = \{X_1,\ldots ,X_n\}$ of *n* random variables and a stochastic function $f:2^A \rightarrow \mathbb {R}$, the goal is to pick a subset $S \subseteq A$ subject to some constraints such that *f*(*S*) is maximized (in expectation). Commonly, the picked subset is constrained by size [31, 32]. There are two types of algorithms for this problem. A *non-adaptive* algorithm decides beforehand which elements to *probe*, i.e. which elements to pick in *S*. A more general *adaptive* algorithm can account for realizations of recent *probes* when making the decision what to *probe* next. This makes *adaptive* algorithms generally stronger than *non-adaptive* ones, but often also substantially more complex and costly. Towards this end, it is of interest to examine the *adaptivity gap*, i.e., the worst-case ratio of the expected objective value of an optimal adaptive and that of a (sub-)optimal non-adaptive algorithm.

**Fig. 1:**
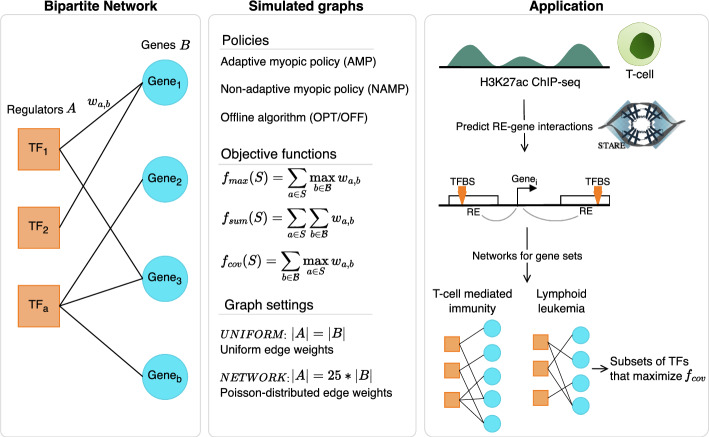
General outline of the study. Transcription factors (TFs) and their target genes are modeled as bipartite networks. Different algorithms, objective functions and graph settings are tested in simulated graphs. In an application to real data, two networks were built based on H3K27ac ChIP-seq data from T-cells. The edges between TFs and genes relied on motif-based transcription factor binding sites (TFBS), for which all predicted regulatory elements (REs) of a gene were considered [36]. The objective was to find sets of TFs with the best coverage of genes involved in T-cell mediated immunity and lymphoid leukemia

In this work, we first simulate TF-gene networks as weighted bipartite graphs with varying settings and test adaptive and non-adaptive algorithms for the choice of a set of TFs (Fig. 1). We evaluate the performance of our algorithms using different objective functions, discuss their properties, and analytically bound their adaptivity gap. While an optimal solution for this task is prohibitively costly to compute, we consider efficient non-adaptive algorithms. Their results compare favorably to more costly adaptive algorithms. In other words, we propose efficient probing algorithms with near-optimal solutions that overcome the issue of testing a prohibitive number of combinations. At its core, our model is equivalent to the classical stochastic probing model [31, 34, 35], since every arbitrary objective function can also be defined in terms of a bipartite graph. However, existing work only formalizes an objective function on a set of random variables. We specialized the model to allow for a clearer and more intuitive formulation, which is especially relevant when using objective functions for which the edges are not independent.

In an application to real data, we construct networks for genes known to be involved in T-cell mediated immunity and genes relevant for lymphoid leukemia. Our objective is to find triplets of TFs with the best network coverage. More precisely, we want to find the combination of TFs with the highest total edge weights in the network, while each gene can only be considered for one TF. The identified TFs show specificity to the gene sets, significant co-expression within the triplets, and we can back up their relevance with literature evidence. Our approach of network construction can easily be applied to different data, since the only required data modality is a sample-specific activity measurement of REs, e.g., ATAC-seq, DNase1-seq or ChIP-seq of RE-associated histone modifications. To our knowledge, there are no other works that optimize the selection of a subset of TFs to maximize the coverage of regulated genes. Also, the probing framework can be applied to TF-gene networks derived from any other available method. In principle, it can also be used on any other kind of bipartite network, for example interactions between REs and the genes they regulate or micro RNAs and their mRNA targets. The code is available on GitHub.

## Methods

### Random graph model

We consider an abstract model to express the regulator probing problem described in the introduction using a graph-theoretic approach. In our model, there is a weighted, complete bipartite graph $G=(A \cup B, A \times B)$ with distinct sets of nodes $\mathcal {A}$ and $\mathcal {B}$. We call the elements of *A* the *regulators* and the elements of *B* *positions*, respectively. We use $n_{\mathcal {A}} :=|\mathcal {A}|$ and $n_{\mathcal {B}} :=|\mathcal {B}|$ to denote their numbers. In our application on biological data, the *regulators* represent TFs and the *positions* represent genes.

In general, every *regulator* $a \in \mathcal {A}$ can influence every *position* $b \in \mathcal {B}$. The extent to which this influence arises is expressed by a *strength* or *edge weight* $w_{a,b} \in [V] = \{0,1,\ldots ,V\}$ for some number $V \in \mathbb {N}$. Here $w_{a,b} = 0$ implies that there is no influence at all. To capture uncertainty in influence, every edge $(a,b) \in \mathcal {A} \times \mathcal {B}$ comes with a discrete distribution $D_{a,b}$ over $\mathcal {V}$. For simplicity, we assume the random weight $w_{a,b}$ is drawn independently according to $D_{a,b}$. Initially, we know $D_{a,b}$, but not the realization $w_{a,b}$.

### Probing and objectives

For our probing problem, we are given two integer parameters *k* and $\ell$, with $\ell \le k \le n_\mathcal {A}$. Our goal is to select a subset $S \subseteq \mathcal {A}$ of at most $\ell$ regulators. We evaluate the quality of this subset *S* by an *objective function* $f:2^A \rightarrow \mathbb {R}_{\ge 0}$. This function depends on the realized edge weights incident to the chosen regulators. As such, we aim to find a set *S* that maximizes it. We say there is a *cardinality constraint of* $\ell$ since only subsets *S* of at most $\ell$ regulators are feasible.

In order to determine a good subset of regulators, we are allowed to *explore* or *probe* up to *k* regulators, where $\ell \le k \le n_\mathcal {A}$. Upon probing a regulator *a*, the realized weights of all incident edges are revealed, i.e., we get to see $w_{a,b}$ for every $b \in \mathcal {B}$. Given the $k \ge \ell$ probed regulators, we can then select a subset *S* of $\ell$ probed regulators that is as good as possible w.r.t. *f*(*S*).

Formally, we express the latter selection step within an extended definition of the objective function, which takes as argument directly the set $S'$ of all *k* probed regulators.

#### Definition 1

For a given objective function $f:2^{\mathcal {A}} \rightarrow \mathbb {R}_{\ge 0}$ with a cardinality constraint of $\ell$, we define its *parent-function* as

$$f^*:2^{\mathcal {A}} \rightarrow \mathbb {R}_{\ge 0}, \qquad S' \mapsto \max _{S \subseteq S':|S| \le \ell } f\left( S\right) .$$

Consequently, our goal is to select a subset $S'$ of size *k* for probing in order to maximize $f^*(S')$.

To model real-life gene regulatory networks, we chose a coverage function as objective function.

#### Definition 2

The *coverage function* is defined by

$$f_{cov}(S) = \sum _{b \in \mathcal {B}} \max _{a \in S} w_{a,b}\hspace{5.0pt}.$$

The name comes from the (weighted) MaximumCoverage problem [37, 38]: after *probing* a subset of *regulators*, we have to select a subset that maximizes the total covered weight where each *position* can only be covered by at most one *regulator*. Therefore, the selected *regulators* depend on each other, which is what we expect in TF-gene networks.

We explore two additional objective functions $f_{max}$ and $f_{sum}$, which treat *regulators* completely independent from each other. These functions are examples of a large class of *regulator additive-separable* functions which we define and analyze in detail in the Supplementary Material.

#### Definition 3

We define

$$f_{max}(S) = \sum _{a \in S}\max _{b \in \mathcal {B}}w_{a,b} \quad \text { and } \quad f_{sum}(S) = \sum _{a \in S}\sum _{b \in \mathcal {B}}w_{a,b}$$

as functions that represent the (sum over all regulators in *S* of the) maximum and the sum of all incident edge weights, respectively.

### Policies and adaptivity gap

Our probing problem can easily be cast as a finite Markov decision process [39]. Consequently, we differentiate between two classes of probing algorithms (often also called probing *strategies* or *policies*): *adaptive* and *non-adaptive*. An adaptive policy takes the *k* probing decisions sequentially using in each step the feedback about realized edge weights from previous steps. In contrast, a non-adaptive one decides upon the complete set $S'$ of *k* probes upfront. Note that in both cases, the final selection of $\ell$ elements from the set of probed elements can be made based on all realized edge weights that were observed during probing.

Any adaptive policy can be described by a decision tree. Since our problem is a finite Markov decision process, there exists an optimal adaptive policy $\pi ^*$. However, such a policy is usually prohibitively costly to compute. Instead, we follow the literature on probing and resort to conceptually and computationally simpler non-adaptive policies. We measure the deterioration in objective value by the standard approach of *adaptivity gap*.

#### Definition 4

Let $\pi ^*$ be an optimal (adaptive) policy and let $\pi '$ be an optimal non-adaptive policy for the probing problem. Suppose $S'_{\pi ^*}$ and $S'_{\pi '}$ are the (random) set of *k* probes by $\pi ^*$ and $\pi '$, respectively. The *adaptivity gap* is given by the ratio of the expected objective function values $\mathbb {E}[f^*(S'_{\pi ^*})] \, / \, \mathbb {E}[f^*(S'_{\pi '})]$.

For the remainder of this article, we refer to policies as algorithms.

### Monotonicity and submodularity

The adaptivity gap in stochastic probing problems has been of significant interest over the last two decades [40–42]. In particular, monotone submodular functions $f^*$ are an important domain that have received significant attention.

#### Definition 5

A non-negative set function $f:2^{\mathcal {A}} \rightarrow \mathbb {R}_{\ge 0}$ is called

- *monotone* if for any two subsets $S \subseteq T \subseteq \mathcal {A}$, we have $f(S) \le f(T)$.
- *submodular* if for any two subsets $S \subseteq T \subseteq \mathcal {A}$ and $j \in \mathcal {A}$, we have $f(T \cup \{j\}) - f(T) \le f(S \cup \{j\}) - f(S)$, i.e., the marginal value of adding an element to a set is diminishing with the size of the set. An alternative definition of submodularity is given by
  $$f(S \cup T) + f(S \cap T) \le f(S) + f(T) \qquad \forall S,T \subseteq A.$$

Notably, in a seminal contribution Asadpour and Nazerzadeh [31] proved that if $f^*$ is monotone submodular, then the adaptivity gap is bounded by $\frac{e}{e - 1} \approx 1.58$. There are cases where this bound is tight. They also provided optimal polynomial-time non-adaptive algorithms to approximate the quality of the optimal (adaptive) algorithm up to a factor arbitrarily close to $\frac{e}{e-1}$.

In our probing problem, we are interested in choosing *k* probes to maximize the parent function $f^*$. Clearly, if the underlying function *f* is monotone, then $f^*$ is also monotone. In contrast to monotonicity, this inheritance is not true for submodularity. We show in the supplement that for every *regulator additive-separable* function $f_{ras}$, the parent function is submodular. In contrast, the coverage function $f_{cov}$ is submodular, but its parent function is not submodular. This motivates the following definition.

#### Definition 6

A function *f* is *parent submodular* if the parent function $f^*$ is submodular.

### Greedy algorithms

Our goal is to design good (non-)adaptive algorithms for regulator probing. Our approach is similar to Asadpour and Nazerzadeh  [31] in the sense that we use greedy algorithms to optimize the different objective functions. We provide an overview over our algorithms, the challenges we face when applying them, and some adjustments as well as consequences for their analysis. Pseudocode of all our algorithms and new results on bounding their approximation guarantees can be found in the Supplementary Material.

Asadpour and Nazerzadeh [31] analyze elementary adaptive (Amp) and non-adaptive (Namp) algorithms that myopically choose the next candidate to probe based on the set of previously chosen regulators. In Amp, the regulator chosen in step *i* is one to maximize the expected marginal increase in the objective function (i.e., value of the best $\ell$ regulators from the previously probed ones and the one in step *i*). Since Amp is adaptive, it can take into account the exact realizations of previous probes, but it relies on expected values for the one chosen in round *i*. Instead, Namp is a non-adaptive version of this algorithm, in which the expected marginal increase is computed over the entire set of all previously chosen regulators and the one in round *i*.

For $f_{max}^*$ and $f_{sum}^*$, we apply Amp without changes after a small precomputation/reduction specified in the Supplementary Material. For Namp and $f^*_{sum}$ (or $f_{max}^*$), we sort regulators by expected sum (or maximum) of incident edge values and probe the *k* regulators with the highest expected values, respectively. This yields a simplification of the marginal increase rule in the original definition of Namp.

When applying these algorithms for regulator probing with objective $f^*_{cov}$, we face multiple challenges. Notably, we observe in the following that $f^*_{cov}$ does not satisfy submodularity. As such, the small constant guarantees on the adaptivity gap from [31] do not readily apply.

#### Proposition 1

$f_{cov}$ is monotone submodular. It is not parent submodular for $k > \ell + 1$.

#### Proof

Monotonicity of $f_{cov}$ and $f_{cov}^*$ is trivial as each *regulator* added to a set *S* can only improve upon the currently obtained value by *S*. Now let $S, T \subseteq \mathcal {A}$. Then,

$$\begin{aligned} f_{cov}\text { is submodular }&\Leftrightarrow f_{cov}\left( S \cup T\right) + f_{cov}\left( S \cap T\right) \le f_{cov}\left( S\right) + f_{cov}\left( T\right) \\&\Leftrightarrow \sum _{b \in \mathcal {B}}\max _{a \in S \cup T}w_{a,b} + \sum _{b \in \mathcal {B}}\max _{a \in S \cap T}w_{a,b} \le \sum _{b \in \mathcal {B}}\max _{a \in S}w_{a,b} + \sum _{b \in B}\max _{a \in T}w_{a,b} \\&\Leftrightarrow \sum _{b \in \mathcal {B}}\left( \max _{a \in S \cup T}w_{a,b} + \max _{a \in S \cap T}w_{a,b}\right) \le \sum _{b \in \mathcal {B}}\left( \max _{a \in S}w_{a,b} + \max _{a \in T}w_{a,b}\right) \\&\Leftarrow \forall b \in \mathcal {B} :\max _{a \in S \cup T}w_{a,b} + \max _{a \in S \cap T}w_{a,b} \le \max _{a \in S}w_{a,b} + \max _{a \in T}w_{a,b}. \end{aligned}$$

Now fix $b \in \mathcal {B}$. Obviously, $\max _{a \in S}w_{a,b}$ as well as $\max _{a \in T}w_{a,b}$ must be greater or equal to $\max _{a \in S \cap T}w_{a,b}$. Furthermore, at least one of $\max _{a \in S}w_{a,b}$ and $\max _{a \in T}w_{a,b}$ must be equal to $\max _{a \in S \cup T}w_{a,b}$, and trivially $\max _{a \in S \cup T}w_{a,b} \ge \max _{a \in S \cap T}w_{a,b}$. Hence, the last inequality holds and $f_{cov}$ is submodular.

To prove that $f_{cov}$ is not *parent-submodular* if $k > \ell + 1$, consider the following instance of our problem:

- We have $$\mathcal {A} :=\{1, 2, 3, 4\}, \mathcal {B} :=\{1, 2, 3, 4\}, \mathcal {V} :=\{0,1\}, k :=4, \ell :=2$$.
- Every $D_{a,b}$ is deterministic meaning that every probability is either 1 or 0 such that we always obtain the realizations
  $$(w_{a,b}) :=\left( \begin{array}{cccc} 1 & 0 & 0 & 0 \\ 0 & 1 & 0 & 0 \\ 0 & 0 & 1 & 0 \\ 1 & 1 & 0 & 1 \end{array}\right) .$$

Consider now the subsets $S \subseteq T \subseteq \mathcal {A}, j \in \mathcal {A}$ with $S = \{1,2\}, T = \{1,2,3\}$ and $j = 4$. Then,

$$\begin{aligned}&f_{cov}^*\text { is not submodular }\\&\Longleftrightarrow \quad f_{cov}^*(T \cup \{j\}) - f_{cov}^*(T)> f_{cov}^*(S \cup \{j\}) - f_{cov}^*(S) \\&\Longleftrightarrow \quad f_{cov}^*(\{1,2,3,4\}) - f_{cov}^*(\{1,2,3\})> f_{cov}^*(\{1,2,4\}) - f_{cov}^*(\{1,2\}) \\&\Longleftrightarrow \quad 4 - 2> 3 - 2 \\&\Longleftrightarrow \quad 2 > 1 \end{aligned}$$

Since we can scale such instances easily, for every $k > \ell + 1$, $f_{cov}^*$ is not submodular.

Moreover, as discussed above, choosing regulators to maximize $f_{cov}$ is a generalization of the (weighted) MaximumCoverage problem [37, 38]. The problem is NP-hard and cannot be approximated within a factor of $\frac{e - 1}{e} - o(1)$, under standard assumptions in computational complexity. Hence, even after we have fully probed *k* elements (non-adaptively or adaptively), we face the problem that the optimal selection of $\ell$ elements is NP-hard to compute. Moreover, in each iteration of the algorithm, we need to evaluate the expected marginal increase in the optimal selection of $\ell$ regulators from an expanded set of probed ones. This again requires solving an instance of the same problem.

An approximation ratio of $\frac{e - 1}{e}$ for our (offline) regulator selection problem can be achieved by a generic greedy algorithm used for the maximization of submodular functions with a cardinality constraint [38]. We term this algorithm Greedy. Our algorithms rely on this procedure to estimate expected marginal gains of probing as well as to make the final selection from the set of probed regulators. More concretely, in Amp, we compute the expected increase in $f^*_{cov}$ using Greedy (starting from the point where we have probed $\ell$ regulators). For that, we iterate over all *unprobed* *regulators*, treat them as *probed* by setting all their incident edge weights to their respective expected values and running Greedy on the now “uncovered” subgraph to estimate the expected marginal increase.

Modifying Namp is not as simple, since the original algorithm relies on re-computations of probability distributions for each position in each iteration. We compute the expected increase precisely for the first $\ell$ rounds, since we know that until this point all regulators would also be selected in the optimal solution. This method, however, fails to remain efficient in subsequent steps due to computational hardness. In later rounds, we instead choose the next regulator as the one with the highest expected sum of incident edge weights. This adjustment can represent a significant departure from the structure of Namp. Unfortunately, showing general approximation factors for this (or any other simple) modification is highly non-trivial due to hardness of the underlying problem. We show approximation factors parametrized by degree in the Supplementary Material, but they significantly underestimate the observed performance in practice. In experiments, we find that the results of this algorithm compare very well against those of the adaptive variant.

To emphasize our modifications, we term the modified algorithms for $f_{cov}^*$ AmpCov and NampCov, respectively. For $f_{max}$ and $f_{sum}$, we still refer to the algorithms by Amp and Namp as we only slightly altered the non-adaptive algorithm and did not alter the adaptive algorithm at all.

Finally, to accurately evaluate these algorithms for $f_{cov}$ in the experiments, one would also have to compute an optimal (offline) selection of regulators for the completely revealed graph. Again, this is infeasible in polynomial time. Instead, we formulate an integer program (IP) (Supp. Figure 10) that can correctly solve such an instance. Unfortunately, even a linear programming (LP) relaxation of this formulation, which can be solved in polynomial time, takes far too long for bigger instances in the Network setting (see below). The original IP itself was already infeasible for smaller instances. Thus, we use the LP-Relaxation to upper-bound the optimal offline value (and thus lower-bound the approximation ratio of our algorithms) in cases where the formulation was runtime-feasible. For other cases, we use Greedy to approximate the optimal offline value and term this application Off.

### Simulated graphs

To explore the practicability of the algorithms discussed above, we ran the adaptive myopic algorithms (Amp/AmpCov) as well as the non-adaptive myopic algorithms (Namp/NampCov) on instances of random graphs. We focused on two scenarios:

1. The Uniform setting: we set $n_{\mathcal {A}} = n_{\mathcal {B}} = |\mathcal {V}| = 16 \cdot z$ for $1 \le z \le 20$. Edge weight distributions $D_{a,b}$ are randomly weighted distributions produced by drawing a random weight in [0, 1] for every possible value the edge-weight can take (and then normalizing all weights).
2. The Network setting: we set $n_{\mathcal {A}} = 16 \cdot z, n_{\mathcal {B}} = 400 \cdot z, |\mathcal {V}| = 10$ for $1 \le z \le 20$. The edge weights model Poisson-distributed random variables with parameter $\lambda \sim [0.5,2.5]$. This setting aims to model gene regulatory networks.

For each *z*, we generated 100 random instances of our random graph model and ran both algorithms 10 times on each graph instance for every $(k,\ell )$ pair with

$$k \in \left\{ \frac{1}{4}n_{\mathcal {A}}, \frac{2}{4}n_{\mathcal {A}}, \frac{3}{4}n_{\mathcal {A}}\right\} , \qquad \ell \in \left\{ \frac{1}{4}k, \frac{2}{4}k, \frac{3}{4}k, \frac{4}{4}k\right\} .$$

The algorithms were then evaluated with the optimal *offline* value $OPT_O$ that could be achieved by choosing $\ell$ *regulators* in this instance, namely an optimal *offline* algorithm Opt. For $f_{cov}$, we used the LP-Relaxation in the (complete) Uniform setting as well as the Network setting for $n_{\mathcal {A}} \le 160$. For bigger instances in the Network setting, we used the offline approximation Off. The pseudocode of the algorithms can be found in the Supplementary Material.

### Computational resources

All algorithms and experiments were implemented in the programming language Rust. The code can be found on GitHub: https://github.com/lukasgeis/BipartiteRegulatorProbing. All experiments were run on a server with an AMD EPYC 7702P 64-Core Processor and 512GB of RAM. To speed up the experiments, instances were generated and evaluated in parallel on up to 64 threads at a time.

### Constructing a TF-gene network

As a real-life application of the algorithms, we constructed a network consisting of TFs (*regulators*) and genes (*positions*) as nodes, with the edge weights representing the regulatory activity that a TF exerts on a gene. This regulatory activity $w_{TF,G}$ was estimated by taking the fraction of all REs of a gene that possess a binding site for the TF:

1 $$\begin{aligned} w_{TF,G}=\frac{|c_{TF}| }{|C_G|} * x {\left\{ \begin{array}{ll} x=1,\ if\ \text {p-value} \le 0.05 \\ x=0,\ \text {otherwise} \end{array}\right. }. \end{aligned}$$

$C_G$ are all REs of a gene, and $c_{TF} \subseteq C_G$ is the subset of REs that contain a predicted binding site for the TF. We require that more REs of a gene have a TFBS than expected in comparison to all REs (p-value $\le$ 0.05 in a Fisher’s exact test, $H_a~=~greater$). If this requirement is not met, $w_{TF,G}$ becomes zero. The contingency table for the enrichment test looks as follows:

**Table:** 

	REs linked to gene G	REs not linked to G
REs with $TFBS_{TF}$		
REs without $TFBS_{TF}$		

The reasoning behind the enrichment requirement was to counteract the imbalanced number of binding sites between TFs. Otherwise, TFs with a high number of motif occurrences would have high edge weights even to genes with an average number of motif-containing REs. All edges with a non-zero edge weight are then assumed to represent a regulatory interaction between a TF and a gene. In other words, in our network a gene is expected to be regulated by all TFs that are connected with the gene.

We built the network for a healthy T-cell sample (IHECRE00000187) from the International Human Epigenome Consortium (IHEC) EpiATLAS (https://ihec-epigenomes.org/epiatlas/data/, metadata version 1.1). To calculate the edge weights, two types of information were required: which REs regulate which genes, and which REs possess which TF binding sites. As candidate REs we used the peaks called on the H3K27ac ChIP-seq signal (MACS2 narrowPeak file [43]). The interactions between the candidate REs and the genes were predicted with the adapted Activity-by-Contact model [36], using the MACS2 signal value as activity and a Hi-C matrix averaged across 35 biosamples as contact data [44]. REs overlapping regions known to accumulate anomalous signal were excluded [45, 46]. The maximum distance between an RE and the transcription start site (TSS) was set to 2.5 MB and the gABC-score cutoff to 0.02. Interactions were predicted for all genes in the GENCODE v38 annotation [47]:
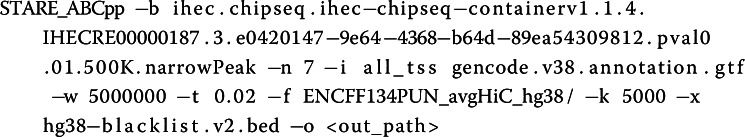


TFBS were annotated with FIMO [48] (v.5.4.1), using a collection of non-redundant motifs from JASPAR 2022 [49], HOCOMOCO v11 [50], and the work of [51] (available under https://github.com/SchulzLab/STARE/blob/main/PWMs/2.2/Jaspar_Hocomoco_Kellis_human_transfac.txt). Only TFs which had a H3K27ac ChIP-seq peak at their promoter ($\pm ~200~bp$ around all annotated TSS) were considered ($n = 420$). For TF dimers all constituent TFs had to have a peak at their promoter. The average base content of the REs was used as background for FIMO. For processing of genomic coordinates we used pybedtools (v0.9.0) [52, 53]. The API of MyGene.info was used for matching gene names to Ensembl IDs [54–56].

We defined two sets of genes, which are more likely to be regulated by similar TFs than the entirety of annotated genes. First, we took all genes that are known to be involved in T-cell mediated immunity from GSEA (biological process ontology GO:0002456) [57, 58]. As second gene set, we collected genes related to lymphoid leukemia by gathering all genes from DISGENET (curated v24.3) [59] that are annotated for any of the following diseases: “*Leukemia, T Cell*”, “*human T cell leukemia*”, “*Early T-Cell Precursor Lymphoblastic Leukemia*”, “*Lymphoid Leukemias*”. We were able to use only genes from the sets that we could find in the gene annotation, leaving 131 genes for T-cell mediated immunity and 83 for lymphoid leukemia.

To find TFs covering the T-cell networks, we used $f_{cov}$ and ran 500 iterations for each $k \in \{30, 40, 50\}$, resulting in a total of 1,500 runs. The number of selected regulators was kept constant at $\ell =3$. In other words, the goal was to find TF triplets with the best network coverage. For each run, the TF triplet with the highest return from $f_{cov}$ was taken. To also test other TF set sizes, we ran 10 iterations each for $3\le \ell \le 10$ while setting $k=50$. OFF was not run for different $\ell$ due to its infeasible runtime for large $\ell$.

To measure co-expression between TFs in a triplet, the expression matrix ’genes_TPM.csv.gz’ from the IHEC EpiATLAS was taken and the correlation between genes assessed with the Pearson correlation coefficient. For each of the three possible pairs of TFs in a triplet, the correlation coefficient was calculated and then averaged to get one value per triplet. In case of TF dimers, the expression values were first averaged across the constituent TFs.

## Results

### Performance on simulated graphs

#### Approximation factors

**Fig. 2:**
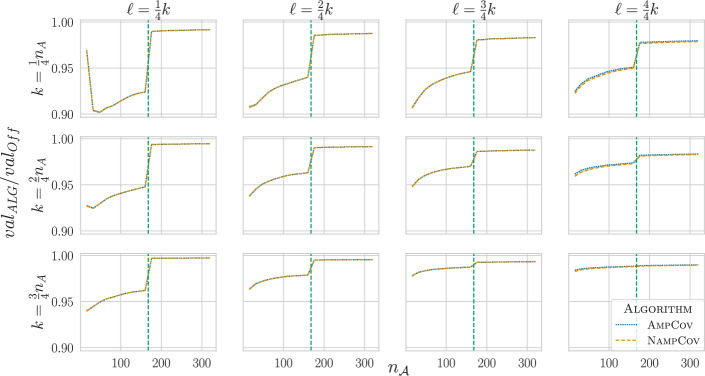
Approximation ratios of AmpCov/NampCov in the Network setting compared to Off for $f_{cov}$ as a function of $n_{\mathcal {A}}$ and $(k,\ell ) \in \left\{ \frac{1}{4}n_A, \frac{2}{4}n_A, \frac{3}{4}n_A\right\} \times \left\{ \frac{1}{4}k, \frac{2}{4}k, \frac{3}{4}k, \frac{4}{4}k\right\}$. The vertical line is the cutoff where Off switched from IP to Greedy

We created multiple graph instances with varying parameters to evaluate the approximation factor of Amp/AmpCov and Namp/NampCov in the Network and Uniform setting while also varying the objective function. In the Network setting with $f_{cov}$, NampCov achieved an almost equal approximation factor compared to AmpCov in all scenarios (Fig. 2). Only for $k = \ell$, where we have an approximation guarantee, the *adaptive* algorithm slightly outperformed the *non-adaptive* one. The average approximation factors ranged from 0.94 to 0.99. For greater $n_{\mathcal {A}}$, we observed better approximation factors because the impact of a single *regulator* on the total value lessens. We also saw a clear trend where *probing more* and *taking less* yields a higher return, since we have more chances at *probing* great regulators without losing value for *probing* bad regulators. The switch from using the LP-Relaxation, which underestimates approximation factors, to the offline greedy approximation, which overestimates approximation factors, is quite visible. However, the bounds for smaller instances and the increasing trend for $n_{\mathcal {A}}$ (towards the step at $n_{\mathcal {A}} = 160$) suggest that regardless of instance size, our algorithms achieve at least $94\%$ of the optimal offline value (which itself is an upper bound for the value of an optimal adaptive policy).

The differences between algorithms were similar for the Uniform setting and the other two objective functions $f_{max}$ and $f_{sum}$ (Supp. Figure 1+2). Compared to $f_{cov}$, the approximation ratios were higher for $f_{max}$ and $f_{sum}$, due to the smaller value space a single edge has.

#### Runtimes

**Fig. 3:**
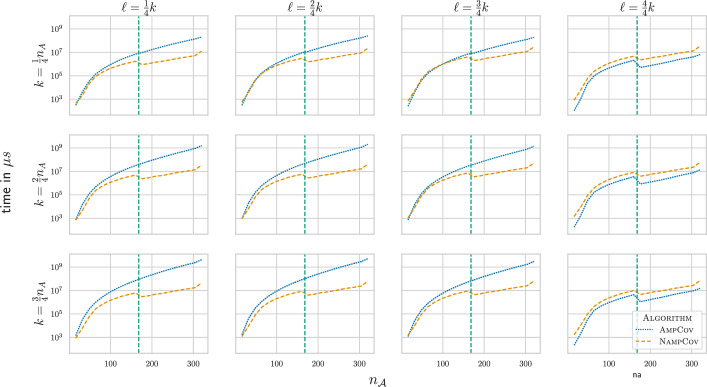
Execution time of AmpCov/NampCov in the Network setting for $f_{cov}$ as a function of $n_{\mathcal {A}}$ and $(k,\ell ) \in \left\{ \frac{1}{4}n_A, \frac{2}{4}n_A, \frac{3}{4}n_A\right\} \times \left\{ \frac{1}{4}k, \frac{2}{4}k, \frac{3}{4}k, \frac{4}{4}k\right\}$. The vertical line is the cutoff where Off switched from IP to Greedy

We compared the runtime of the algorithms, objective functions, and graph settings on the simulated graphs (Fig. 3, Supp. Figure 3–5). Overall, for $\ell < k$, NampCov was always faster than AmpCov. Due to the need to run the greedy algorithm of Nemhauser et al. [38] multiple times in each *probing* phase after $\ell$ *probes* in the *adaptive* algorithm, AmpCov was far slower in the Network setting for $\ell < k$ with $f_{cov}$. However, for $\ell = k$, AmpCov ran faster than Namp in both settings, since we do not use the greedy approximation anymore as a subroutine. The slight dropoff in runtime after the cutoff is probably a byproduct of different scheduling on the server as we ran these two parts of the experiment at different times. This, however, has no effect on our observations.

For the other two objective functions $f_{max}$ and $f_{sum}$ (Supp. Figure 3+4), every algorithm needed less than a hundredth of a second which leads to a lot of noise in the timings which are possibly further enhanced by the introduced parallelism.

### Identification of TFs covering T-cell networks

**Fig. 4:**
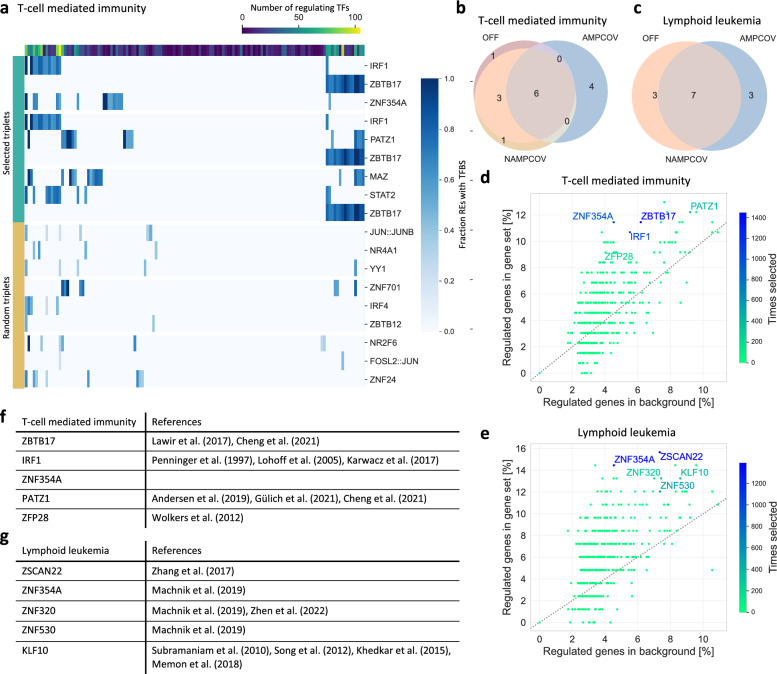
Application of the algorithms to find transcription factors (TFs) in T-cell networks. **a** Coverage of the genes in the T-cell mediated immunity network for three of the most frequently chosen TF triplets by NampCov compared to three randomly selected TF triplets. The top TF triplets were selected from the top 10 and picked to reduce redundancy of individual TFs. Each column represents one gene of the network. The first row shows the number of TFs that have a non-zero edge to a gene, i.e. TFs with an enrichment of transcription factor binding sites (TFBS) in a gene’s REs (*p*-value $\le ~0.05$). The fraction of a gene’s regulatory elements (REs) with TFBS represent the edge weights of the network. For the coverage of the lymphoid leukemia network as well as visualizations of the full networks see Supp. Figure 6a and Supp. Figure 7+8. **b**, **c** Venn diagrams of the overlap of the ten most frequently chosen TF triplets by the three algorithms for the networks. **d**, **e** Percentage of all annotated genome-wide genes that are regulated by a TF (Background regulation) versus the percentage of regulated genes from the respective gene set. Only genes with at least one RE were considered. The color shows the number of times a TF was selected by NampCov. Top five TFs ranked by the number of times they were within a triplet selected by NampCov for both networks along with literature references supporting the TFs’ function in T-cell mediated immunity (**f**) and lymphoid leukemia (**g**)

For a biological application, we used the probing algorithms on networks consisting of TFs and genes, for which the data originated from a T-cell sample. A network was constructed for each of two gene sets: genes being relevant for T-cell mediated immunity and genes reported to be involved in lymphoid leukemia. In our setup, the algorithms were run repeatedly to choose sets of three TFs to maximize the objective function $f_{cov}$. We also tested larger sets, but mainly focused on TF triplets. As expected, the most frequently chosen TF triplets regulated more genes of the networks than randomly assembled TF triplets (Fig. 4a, Supp. Figure 6a). However, for a part of the genes no regulating TF was chosen. Especially genes that are connected to only very few TFs were rarely included, suggesting that even within predefined gene sets the regulatory programs are diverse and therefore difficult to capture with only three TFs. More genes can be covered by increasing the TF set size, but even a set of size 10 covered only $53\%$ of the T-cell mediated immunity genes and $65\%$ of the lymphoid leukemia genes (Supp. Figure 6b+c). The chosen sets of size three were assembled mostly from similar TFs with little variation across the top ranked TF triplets (Supp. Figure 6d, e). The most frequently chosen TF triplets were similar between Off and NampCov, while AmpCov selected a few distinct TF triplets (Fig. 4b, c). The results across the three tested choices for *k* (number of TFs that can be probed) were similar, especially for AmpCov and NampCov which produced almost identical results (Supp. Figure 6f). We also tested varying thresholds for the network construction, i.e. when an edge is drawn between a gene and a TF. As expected, more lenient cutoffs lead to a higher number of genes that are targeted by a TF (Supp. Figure 9a–d). Since the change in cutoffs varies the ranking between TFs in terms of the number of connected genes (Supp. Figure 9b, e), the prioritization of the probing algorithm also changes accordingly (Supp. Figure 9c+f). However, unless too many TF-gene edges are removed, the results remain stable.

To explore the relation of the TFs within a triplet, we investigated the co-expression between TFs based on the RNA-seq data of the IHEC EpiATLAS (1555 samples). For all algorithms and both networks, the most frequently selected TF triplets had a significantly higher co-expression among the individual TFs than random TF triplets (*p*-value $\le$ 0.05, two-sided Mann–Whitney U-Test, Supp. Figure 6 g, h). This hints towards similar regulatory programs of the TFs in a triplet.

To evaluate the individual TFs, we ranked them by how often they were present in the selected TF triplets. For this, we focused on NampCov, since this algorithm is the most efficient while still coming close to the optimal solution on simulated networks. We evaluated how specific the most frequently represented TFs are, by comparing the percentage of genes in the gene sets with which a TF is connected in the network to the percentage from a genome-wide background. The background consisted of all annotated genes with at least one RE. For both tested networks, NampCov selected TFs that regulate more members of the gene sets relative to the background (Fig. 4d+e). This effect is data-driven, since the algorithms were exclusively run on the gene set-specific networks and did not use any knowledge about the background. Nonetheless, it indicates the specificity of the prioritized TFs and hints towards distinct regulatory modules of the gene sets. For many of the selected TFs, their involvement in T-cell mediated immunity and lymphoid leukemia was already reported in the literature (Fig. 4f, g). One frequently selected TF was ZBTB17, which, besides its importance for early embryonic development, was also found to be involved in lymphoid development and the specification of T-cell phenotypes [60, 61]. IRF1 was reported to be required for the differentiation and function of specific T-cell subtypes [62–64]. Another frequently selected TF for genes of T-cell mediated immunity was PATZ1, for which multiple studies showed its importance in the development of different T-cell populations [61, 65, 66]. Multiple TFs that were repeatedly selected from the network of lymphoid leukemia genes were found to be differentially expressed in various cancer samples of the TCGA atlas [67], including ZNF354A, ZNF530 and ZNF320. ZNF320 was additionally found to be associated with immune infiltration in hepatocellular carcinoma [68]. ZSCAN22 was predicted to be a target of micro RNAs that were associated with the survival of patients with acute myeloid leukemia [69]. Another frequently chosen TF was KLF10, which has been repeatedly found to be a tumor suppressor in various cancer types [70–72], and to regulate the differentiation of T-cell subtypes [73]. For some of the reported TFs, the literature support was sparse or relatively indirect, which might be explained by many of the TFs being understudied and consequently a lack of known functions.

Overall, we were able to apply the method to a biological data set and found meaningful sets of TFs that are likely regulating gene sets with specific functions in T-cells.

## Discussion and conclusion

We formulated the challenge of finding a set of TFs in a regulatory network that maximize the impact on affected genes with the probing problem. We evaluated different objective functions, and showed different approximation guarantees in distinct cases. On simulated graphs, we found that a non-adaptive greedy algorithm could perform similarly well as an adaptive greedy one, while often being faster to compute. While we found that more trivial objective functions such as $f_{max}$ and $f_{sum}$ easily uphold approximation guarantees of Asadpour and Nazerzadeh [31], more intricate and realistic objective functions such as $f_{cov}$ fall short of necessary requirements. Even for reasonably sized instances, solving the offline problem using an LP-Relaxation is quickly infeasible. We thus used modified greedy algorithms that perform very well in practice for $f_{cov}$. We also proved performance guarantees for the unmodified versions of the algorithms based on the maximum degree. More concretely, we showed that general objective functions that view genes as independent incur a loss proportional to the maximum gene node-degree in the approximation guarantee when applying algorithms from Asadpour and Nazerzadeh . For $f_{cov}$, we could also show that this loss is proportional to the global maximum degree in the network (TF or gene).

In an application of the algorithms on networks built from T-cell data, we were able to find meaningful triplets of TFs with known functions in regulating the respective gene sets. Unlike other methods that examine TF-gene relationships, we consider all potential REs of a gene and did not restrict it to the promoter region. A few caveats have to be considered with regard to the construction of the network. We used position weight matrices to find TFBS, which is a simplified model for TF binding that considers the individual binding positions to be independent. Furthermore, TFBS were binarized, which disregards potential influence of low-affinity binding sites [2]. Motif-based TFBS prediction also introduces the challenge of comparing TFs with redundant binding preferences. Further, we modeled the regulatory strength between a TF and a gene by the fraction of REs that a TF binds, with the requirement that bound REs had to be enriched. This estimate of regulatory strength is unlikely to fully capture a TF-gene relationship, as the frequency of TF binding is not expected to translate linearly into expression regulation. Multiple other unaccounted factors may be involved, such as presence of cofactors, different modes of cooperation between TFs, post-translational modifications or chromatin modifications [2, 74, 75]. Importantly, the networks used in this work serve as example for applying the probing algorithms. They can be replaced by other TF-gene networks constructed with any of the available methods or databases [4–7, 76]. The only requirements are that they are built as bipartite graph, meaning there are only edges between TFs and genes, and that the edge weights are positive. In the context of designing perturbation experiments, our selection of TF subsets is optimized for the particular gene set without knowledge about the genome-wide network. As consequence, the TFs do not have to be specific to the gene set but can potentially also regulate other genes. Nonetheless, in our application the selected TFs showed specificity for the particular gene sets, which could be owed to genes with similar functions being more likely to be targeted by similar TFs. Another point to consider is that all genes in our setup are weighted equally. In certain scenarios it might be desirable to weigh genes differently, e.g. for genes of a pathway that those at central positions should be more important than those further downstream. This could be implemented by scaling the edge weights according to the gene’s importance.

The minimal data requirements of our approach, namely a measurement of RE activity, enables it to be readily applied to other data. The identification of a set of TFs with the highest coverage in TF-gene networks could serve as a starting point for finding candidates for perturbation studies. The size of the TF set is flexible and can be chosen depending on the question at hand. An interesting direction for the future is the application of the algorithms on other types of networks, for example interactions between micro RNAs and their mRNA targets, or ligand-receptor networks.

## Additional file

**Additional file 1.** Supplemental Materials and Methods.

## Data Availability

The code for the simulated graph experiments, as well as for the real-data application, and scripts to generate TF-gene networks for custom data is available under https://github.com/lukasgeis/BipartiteRegulatorProbing release v0.1. There, we also provide the two T-cell networks on which the results of the biological application are based. The data from which the networks were generated is available via the International Human Epigenome Consortium (IHEC) EpiATLAS (IHECRE00000187, https://ihec-epigenomes.org/epiatlas/data/).
