# Supplementary material for: Probing transcription factor subsets in gene regulatory networks
Source: Algorithms Mol Biol. 2026 May 15;21:7. doi: 10.1186/s13015-026-00297-x (PMC13179610; doi:10.1186/s13015-026-00297-x)
Supplement: Supplementary file 1 — Additional file 1. Supplemental Materials and Methods. [file 13015_2026_297_MOESM1_ESM.pdf]

Supplementary Material: Probing transcription factor subsets in gene regulatory networks

1 Regulator Additive-Separable Functions

In this section, we analyze functions that are regulator additive-separable and redefine two such functions (f_{max} and f_{sum}) as objective functions for our probing problem.

Definition 1. *A function* $f_{ras}\colon 2^{\mathcal{A}} \to \mathbb{R}_{\geq 0}$ *is called* regulator additive-separable *if*

$$f_{ras}(S) = \sum_{a \in S} v(w_a) \ ,$$

where $v(w_a)$ *is an arbitrary non-negative function of the vector* $w_a = (w_{a,b})_{b \in \mathcal{B}}$ *of edge weights incident to* $a \in \mathcal{A}$*.*

Definition 2. *We define two regulator additive-separable functions. For a subset* $S \subseteq \mathcal{A}$ *of regulators, they consider the maximum and the sum of all incident edge-weights, respectively:*

$$f_{max}(S) = \sum_{a \in S} \max_{b \in \mathcal{B}} w_{a,b} \quad \text{and} \quad f_{sum}(S) = \sum_{a \in S} \sum_{b \in \mathcal{B}} w_{a,b}.$$

The key insight is that these functions allow us to reduce the probing problem to a standard scenario that we term the Top-ℓ-ProbeMax problem [1, 2]. In this problem, we select k boxes to be probed. Each box contains a non-negative prize drawn from an individual, independent distribution. Given the set of k probed boxes, we then choose the $\ell \leq k$ boxes with the highest prizes among them.

Indeed, we can interpret the set of regulators $\mathcal{A}$ as the boxes, and the aggregated value $v(a)$ as the prize in box $a \in \mathcal{A}$. Clearly, by analyzing the distributions $(D_{a,b})_{b \in \mathcal{B}}$, we can obtain a distribution D_a for the aggregated value $v(a)$. For completeness, we show that the objective function is parent submodular and, as such, the results from [3] are applicable.

Proposition 1. Regulator additive-separable *objective functions (and, thus, the objective in* Top-ℓ-ProbeMax*) are parent-submodular.*

Proof. Consider an arbitrary *regulator additive-separable* objective function f_{ras} and its corresponding *parent-function*

$$f^*_{ras}\colon 2^{\mathcal{A}} \to \mathbb{R}_{\geq 0}, \qquad S \mapsto \max_{S' \subseteq S\colon |S'| \leq \ell} \sum_{a \in S'} v(a)$$

Now let $S \subseteq T \subseteq \mathcal{A}$ and $j \in \mathcal{A}$. For $|T| < \ell$, trivially $f^*_{ras}(T \cup \{j\}) - f^*_{ras}(T) \leq f^*_{ras}(S \cup \{j\}) - f^*_{ras}(S)$, because if $j \in S$, then both sides equal 0 and if $j \notin S$, then the right side is $v(j) > 0$ and the left side either evaluates to 0 or $v(j)$ depending on whether $j \in T$ or $j \notin T$.
Thus, let $|T| \geq \ell$. For a set $A \subseteq \mathcal{A}$, let A^{max}_i denote the ith-largest element in A. Then,

$$\begin{aligned}
& f^*_{ras} \text{ is submodular} \\
\iff\ & f^*_{ras}(T \cup \{j\}) - f^*_{ras}(T) \quad \leq \quad f^*_{ras}(S \cup \{j\}) - f^*_{ras}(S) \\
\iff\ & \max_{T' \subseteq T \cup \{j\}: |T'| \leq \ell} \sum_{a \in T'} v(a) - \max_{T' \subseteq T: |T'| \leq \ell} \sum_{a \in T'} v(a) \\
& \leq \quad \max_{S' \subseteq S \cup \{j\}: |S'| \leq \ell} \sum_{a \in S'} v(a) - \max_{S' \subseteq S: |S'| \leq \ell} \sum_{a \in S'} v(a) \\
\iff\ & \max\{j - T^{max}_\ell, 0\} \quad \leq \quad \max\{j - S^{max}_\ell, 0\}
\end{aligned}$$

where the last equivalence follows because for every subset $A \subseteq \mathcal{A}$, the value of $f^*_{ras}(A)$ is the sum of the ℓ highest $v(a)$ of $a \in A$. Hence, if $|A| \geq \ell$ and another *regulator* j is added to A, then f^*_{ras} either increases by $v(j) - A^{max}_\ell$ if $v(j)$ is greater than A^{max}_ℓ or does not increase if it is smaller. Additionally, the last inequality is true, because clearly $T^{max}_\ell \geq S^{max}_\ell$ since $T \supseteq S$ and f^*_{ras} is a monotone function. □

We can use Amp by greedily and adaptively *probing* the regulator which yields the highest expected increase in our objective function. This leads to a $\left(1 - \frac{1}{e}\right) \cdot OPT_A$ approximation, where OPT_A is the optimal adaptive algorithm for f_{ras}. For the *non-adaptive* variant, we simplify Namp by greedily *probing* the regulators which have the highest a-priori expected reward.

Symmetrically, we consider objectives that are additive separable over *positions* which are significantly harder to handle as seen by f_{cov}.

Definition 3. *A function* $f_{pas}\colon 2^{\mathcal{A}} \to \mathbb{R}_{\geq 0}$ *is called* position additive-separable *if*

$$f_{pas}(S) = \sum_{b \in \mathcal{B}} v(w_b) \ ,$$

where $v(w_b)$ *is an arbitrary non-negative function of the vector* $w_b = (w_{a,b})_{a \in S}$ *of edge weights incident to* $b \in \mathcal{B}$.

We note that f_{sum} can also be classified as a *position additive-separable* function.

2 Bounded Degree

In this section, we explore algorithms parameterized by the *degree* of *regulators* or *positions*. More formally, the degree of a regulator $a \in \mathcal{A}$ is the number of edges (a, b), $b \in \mathcal{B}$, for which $\Pr[w_{a,b} > 0] > 0$. We term such edges 1-edges. The degree of position $b \in \mathcal{B}$ is defined analogously. We find it convenient to drop from consideration all edges with $\Pr[w_{a,b} = 0] = 1$ which we term 0-edges.

Note that this parameterization also implies that any objective function can only depend on non-zero edges and thus $v(0) = 0$. Let $\Delta_{\mathcal{A}}$ denote the maximum degree of any *regulator* and $\Delta_{\mathcal{B}}$ the maximum degree of any *position.*

Bounded Position-Degree

For instances with bounded $\Delta_{\mathcal{B}}$, we show the following result:

Theorem 2. *For monotone* position additive-separable *objective functions f_{pas}, algorithms of [3] only lose an additional factor of $\frac{1}{\Delta_{\mathcal{B}}}$.*

To prove this theorem, consider the following algorithm extension:

1. For any *position* $b \in \mathcal{B}$, pick one of its incident edges independently uniformly at random. Let that edge be e_b.
2. Consider the subgraph G' which only consists of edges e_b for all $b \in \mathcal{B}$.
3. Run any (*adaptive* or *non-adaptive*) algorithm $\mathcal{P}$ on G' to *probe* a subset S of k and choose a subset of ℓ *regulators.*

Lemma 3. *Restricting to G' only incurs a loss of $\frac{1}{\Delta_{\mathcal{B}}}$ in the optimal algorithm.*

Proof. W. l. o. g. assume that every *position* has exactly $\Delta_{\mathcal{B}}$ incident edges (otherwise count a few 0-edges). Now, for every *position*, number its incident edges arbitrarily from 1 to $\Delta_{\mathcal{B}}$ and split G into edge-disjoint subgraphs $G^1, \dots, G^{\Delta_{\mathcal{B}}}$ where G^i only contains edges that are numbered i. Consider the optimal (deterministic and *adaptive*) algorithm $\textsc{Opt}$ in G. Let $v_{\textsc{Opt}}(G)$ denote the expected reward of $\textsc{Opt}$ in G and let $v_{\textsc{Opt}}(G^i)$ denote the expected value of the edges that are present in G^i and are chosen by $\textsc{Opt}$ in G in the final coverage. Since $G^1, \dots, G^{\Delta_{\mathcal{B}}}$ are edge-disjoint, clearly,

$$v_{\textsc{Opt}}(G) = \sum_{i=1}^{\Delta_{\mathcal{B}}} v_{\textsc{Opt}}(G^i).$$

However, only counting edges in G^i is a subproblem and thus also a viable instance. Hence, for each G^i, there must exist an optimal (*adaptive*) algorithm $\textsc{Opt}^i$ that maximizes f^*_{pas} in G^i. Let $v_{\textsc{Opt}^i}(G^i)$ denote the expected reward of such algorithm. Because $\textsc{Opt}^i$ is optimal in G^i, we must have $v_{\textsc{Opt}}(G^i) \leq v_{\textsc{Opt}^i}(G^i)$ for every i and thus

$$v_{\textsc{Opt}}(G) \leq \sum_{i=1}^{\Delta_{\mathcal{B}}} v_{\textsc{Opt}^i}(G^i).$$

Since we pick an incident edge for every *position* in step (1) uniformly at random, this is equivalent to uniformly setting $G' := G^i$ and hence the expected reward of the optimal (*adaptive*) algorithm in G' can at most be a fraction of $\frac{1}{\Delta_\mathcal{B}}$ of the reward of the original optimal (*adaptive*) algorithm in G. ◻

Lemma 4. *The subproblem in step (3) can be reduced to the* Top-ℓ-ProbeMax *problem.*

Proof. Consider f_{pas} in G'. For every *position* $b \in \mathcal{B}$, we have exactly one incident edge (a_b, b) in G' that is not a 0-edge. Hence, the term $v(w_b)$ is equivalent to $\sum_{a \in S} v(w_{a,b})$ since there is at most one *regulator* in S with a non-zero edge to b and $v(0) = 0$ as stated earlier. Thus, in G', we have

$$f_{pas}(S) = \sum_{b \in \mathcal{B}} v(w_b) = \sum_{b \in \mathcal{B}} \sum_{a \in S} v(w_{a,b}) = \sum_{a \in S} \left(\sum_{b \in \mathcal{B}} v(w_{a,b}) \right) = \sum_{a \in S} v'(w_a)$$

where $v'(w_a) = \sum_{b \in \mathcal{B}} v(w_{a,b})$. Since $v(\cdot)$ is a non-negative function, $v'(\cdot)$ also is and therefore can be reduced to Top-ℓ-ProbeMax. ◻

Proof of Theorem 2. Clearly, using the results of Lemma 3 and Lemma 4, algorithms of Asadpour and Nazerzadeh [3] with an approximation factor α achieve an $\frac{\alpha}{\Delta_\mathcal{B}}$ approximation of the optimal adaptive algorithm in G when used in G'. Since algorithms of Asadpour and Nazerzadeh are greedy algorithms and f_{pas} is assumed to be monotone, every *probe* in G is at least as good as the corresponding *probe* in G'. Hence, we do not lose an additional factor if we use the algorithm in G instead of G', thus maintaining the approximation factor of $\frac{\alpha}{\Delta_\mathcal{B}}$. ◻

Bounded Regulator-Degree

For bounded $\Delta_\mathcal{A}$, we can prove a similar result for f_{cov}.
Theorem 5. *For f_{cov}, algorithms of [3] only lose an additional factor of $\frac{1}{\Delta_\mathcal{A}}$.*

Proving Theorem 5 in the general case is unfortunately significantly harder as we require f_{pas} (and subsequently $v(w_b)$) to be *parent-submodular* which is a non-trivial property to the best of our knowledge.

We prove this theorem with a similar algorithm extension to the one before:

1. For any *regulator* $a \in \mathcal{A}$, pick one of its incident edges independently uniformly at random. Let that edge be e_a.
2. Consider the subgraph G' which only consists of edges e_a for all $a \in \mathcal{A}$.
3. Run any (*adaptive* or *non-adaptive*) algorithm $\mathcal{P}$ on G' to *probe* a subset S of k and choose a subset of ℓ *regulators*.

Lemma 6. *Restricting to G' only incurs a loss of $\frac{1}{\Delta_\mathcal{A}}$.*

Proof. The proof is analogous to the proof of Theorem 2. ◻

Lemma 7. *The subproblem in step (3) is parent-submodular.*

Proof. Consider f^*_{cov} in G'. For every *regulator* $a \in \mathcal{A}$, we have exactly one incident edge in G' that is not a 0-edge. Hence, every *regulator* can influence at most one *position*. Now let $S \subseteq T \subseteq \mathcal{A}$ and $j \in \mathcal{A}$. We want to show that

$$f^*_{cov}(T \cup \{j\}) - f^*_{cov}(T) \leq f^*_{cov}(S \cup \{j\}) - f^*_{cov}(S).$$

Let S_{cov}, T_{cov} and S^j_{cov}, T^j_{cov} denote the subsets of at most ℓ *regulators* chosen by $f^*_{cov}(S), f^*_{cov}(T)$ and $f^*_{cov}(S \cup \{j\}), f^*_{cov}(T \cup \{j\})$ respectively where we do not include 0-edges. Let $b_j \in \mathcal{B}$ denote the position to which j has its 1-edge. If $j \notin T^j_{cov}$, then the left side of the equation is 0, hence proving the theorem due to the monotonicity of f^*_{cov}.

Hence, assume that $j \in T^j_{cov}$. If $|T| < \ell$, then the statement follows due to the submodularity of f_{cov}. Thus, also assume $|T| \geq \ell$. Again, due to monotonicity and submodularity, the statement trivially follows if $|S| < \ell$. Therefore, also assume $|S| \geq \ell$.

If there was another *regulator* $i \in T_{cov}$ with its 1-edge connected to b_j, then the left side of the equation equals $w_{j,b_j} - w_{i,b_j} \geq 0$ since every other edge in T_{cov} is at least as good as (i, b_j). Because every position can only have one incident *active* edge in f^*_{cov}, we either have $i \in S_{cov} \backslash S^j_{cov}$ and hence equality in the equation or $m \in S_{cov} \backslash T_{cov}$ for some $m \in \mathcal{A}$ with $w_{m,b_j} \geq 0$. But then, again due to the monotonicity of f^*_{cov}, we must have $w_{i,b_j} > w_{m,b_j}$ because $m \notin T_{cov}$ and hence the right side must be greater than the left side of the equation. The only case where the left side could be greater than the right side is when there does not exist such i in T_{cov} but in S_{cov} with $w_{i,b_j} > 0$. But then, at some point in the algorithm, we must have replaced i in the chosen set because we have found at least k *regulators* which had a higher value on their 1-edge. Consequently, when probing j, we must already have chosen k *regulators* in T and thus replace some $m \in T_{cov}$ with value w_{m,b_m} with j. The left side hence equals $w_{j,b_j} - w_{m,b_m} > 0$. But since we substituted i earlier, we know that $w_{m,b_m} > w_{i,b_j}$, thus making the right side of the equation greater and proving the theorem. □

Proof of Theorem 5. The theorem follows using the same arguments as in the proof of Theorem 2 using Lemma 6 and Lemma 7. □

3 Results

3.1 Simulated Graphs

Approximation Factors

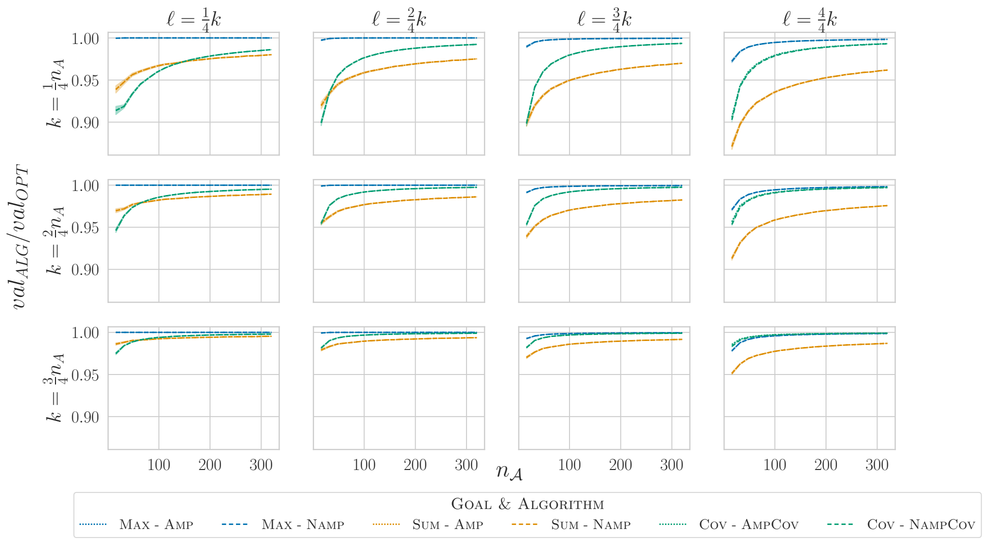

Fig. 1 Approximation ratios of Amp/AmpCov and Namp/NampCov in the Uniform setting compared to Opt/Off for $f_{max}, f_{sum}, f_{cov}$ as a function of $n_{\mathcal{A}}$ and $(k, \ell) \in \{\frac{1}{4}n_A, \frac{2}{4}n_A, \frac{3}{4}n_A\} \times \{\frac{1}{4}k, \frac{2}{4}k, \frac{3}{4}k, \frac{4}{4}k\}$.

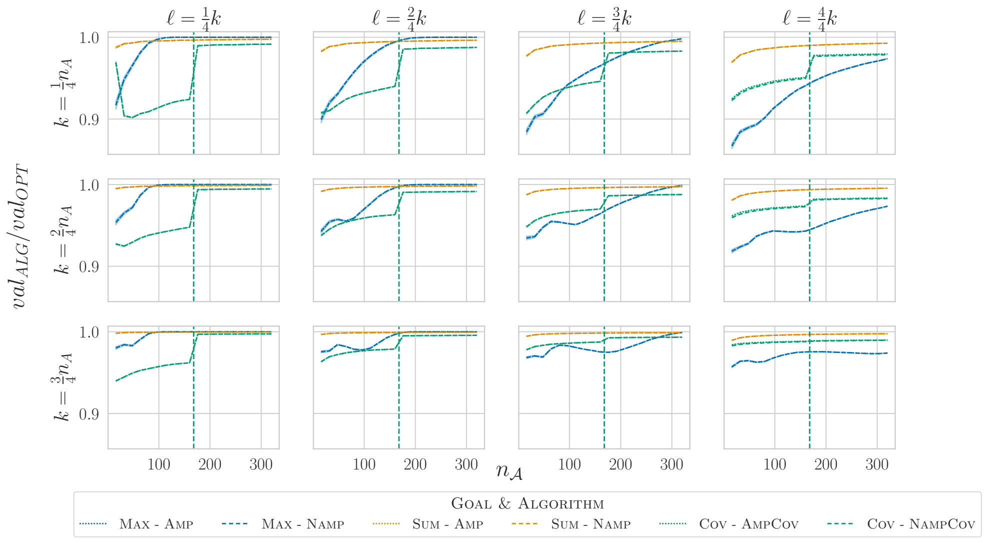

Fig. 2 Approximation ratios of Amp/AmpCov and Namp/NampCov in the Network setting compared to Opt/Off for $f_{max}, f_{sum}, f_{cov}$ as a function of $n_{\mathcal{A}}$ and $(k, \ell) \in \{\frac{1}{4}n_A, \frac{2}{4}n_A, \frac{3}{4}n_A\} \times \{\frac{1}{4}k, \frac{2}{4}k, \frac{3}{4}k, \frac{4}{4}k\}$. The vertical line is the cutoff where Off switched from IP to Greedy for f_{cov}.

Runtime

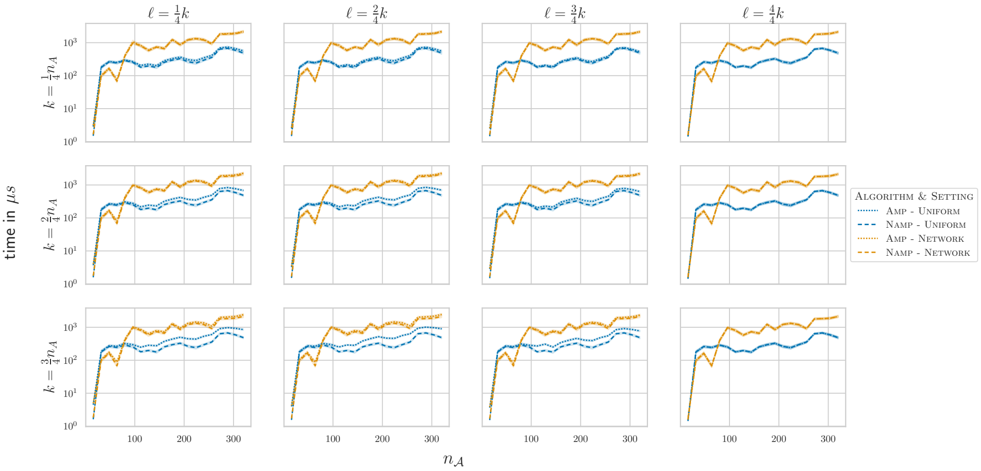

Fig. 3 Execution time of Amp/Namp in the Uniform and Network setting for f_{max} as a function of $n_{\mathcal{A}}$ and $(k, \ell) \in \left\{\frac{1}{4}n_{\mathcal{A}}, \frac{2}{4}n_{\mathcal{A}}, \frac{3}{4}n_{\mathcal{A}}\right\} \times \left\{\frac{1}{4}k, \frac{2}{4}k, \frac{3}{4}k, \frac{4}{4}k\right\}$. The plots show error bands showing the 95% confidence interval across all iterations.

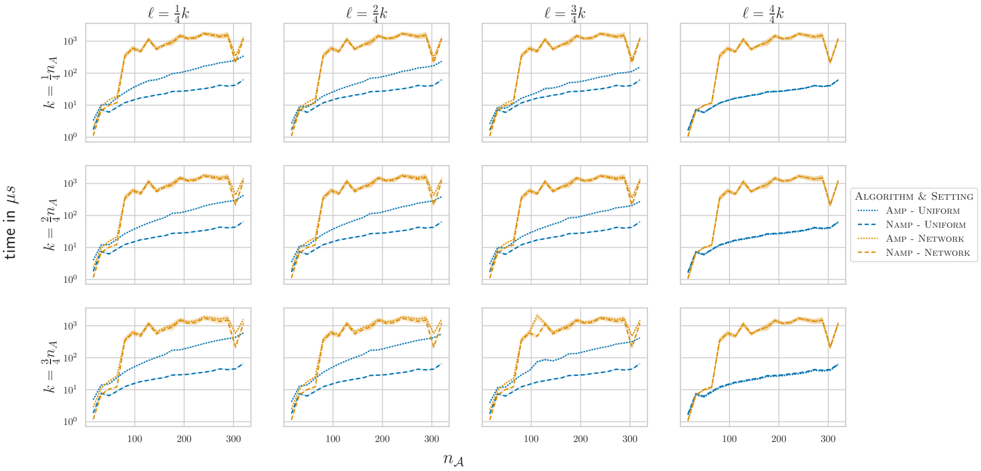

Fig. 4 Execution time of Amp/Namp in the Uniform and Network setting for f_{sum} as a function of $n_{\mathcal{A}}$ and $(k, \ell) \in \left\{\frac{1}{4}n_{\mathcal{A}}, \frac{2}{4}n_{\mathcal{A}}, \frac{3}{4}n_{\mathcal{A}}\right\} \times \left\{\frac{1}{4}k, \frac{2}{4}k, \frac{3}{4}k, \frac{4}{4}k\right\}$. The plots show error bands showing the 95% confidence interval across all iterations.

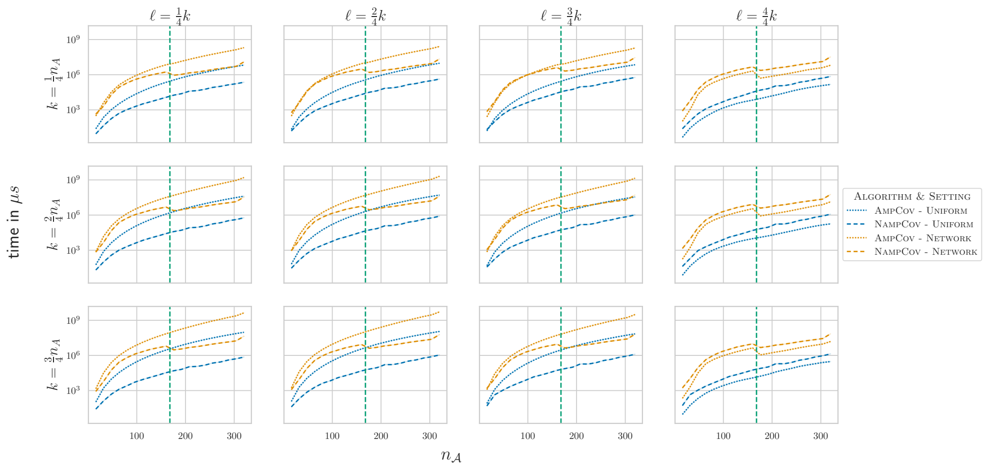

Fig. 5 Execution time of AmpCov/NampCov in the Uniform and Network setting for f_{cov} as a function of $n_{\mathcal{A}}$ and $(k, \ell) \in \{\frac{1}{4}n_A, \frac{2}{4}n_A, \frac{3}{4}n_A\} \times \{\frac{1}{4}k, \frac{2}{4}k, \frac{3}{4}k, \frac{4}{4}k\}$. The plots show error bands showing the 95% confidence interval across all iterations. The vertical line is the cutoff where Off switched from IP to Greedy.

3.2 TF-Gene Networks

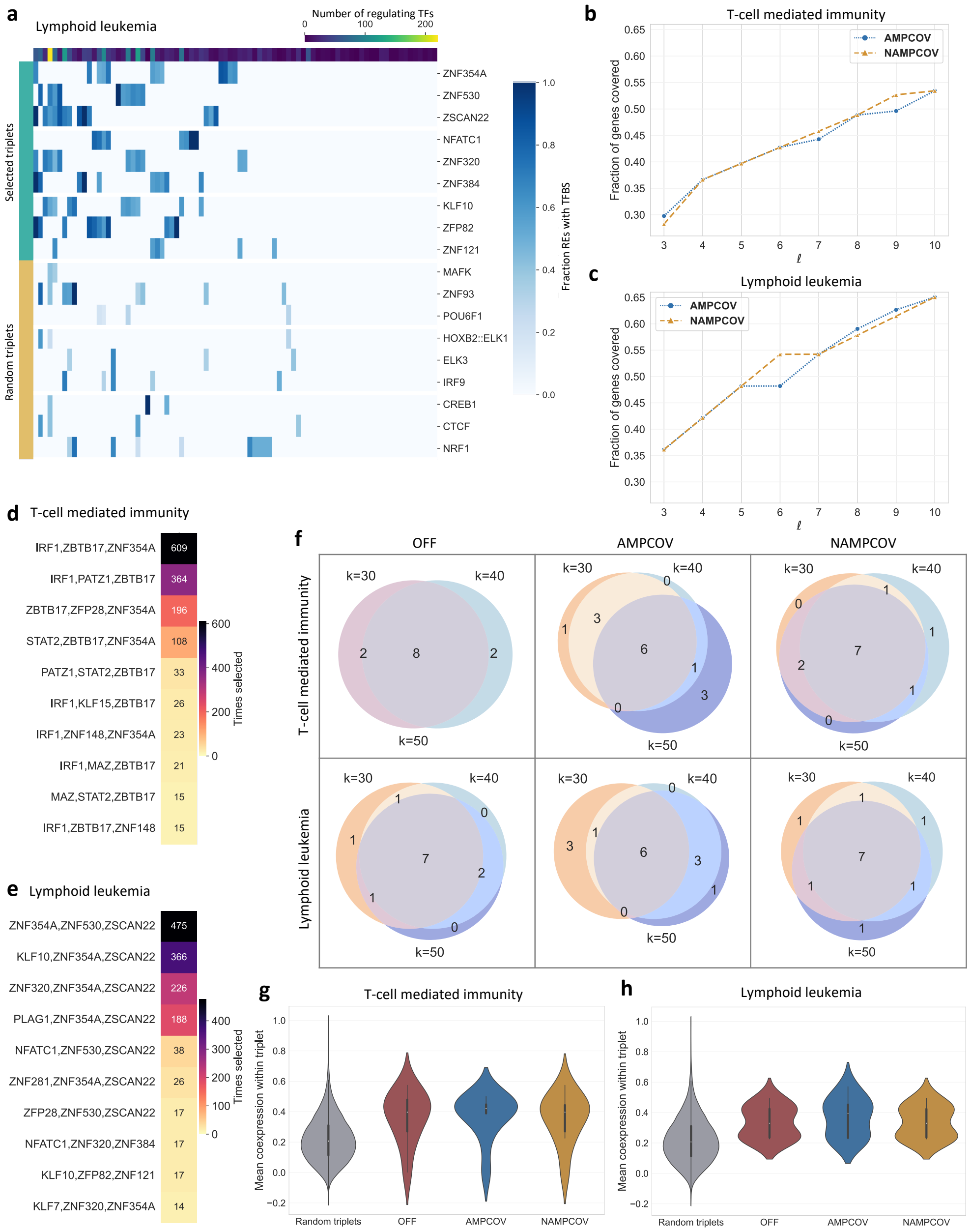

Fig. 6 Transcription factor (TF) triplets chosen by the algorithms to cover T-cell networks. (a) Coverage of the genes in the lymphoid leukemia network for the three most frequently chosen TF triplets by NampCov compared to three randomly selected TF triplets. The top TF triplets were selected from the top 10 and picked to reduce redundancy of individual TFs. Each column represents one gene of the network. The first row shows the number of TFs that regulate a gene. The fraction of a gene's regulatory elements (REs) with transcription factor binding sites (TFBS) represent the edge weights of the network. (b+c) Fraction of genes covered (≥ 1 non-zero edge) by TF sets of varying size (ℓ), selected by AmpCov and NampCov. For each ℓ, the set that was chosen most often across 10 iterations with $k = 50$ was taken. (d+e) Top 10 most frequently selected TF triplets for the T-cell mediated immunity (d) and lymphoid leukemia (e) network. (f) Overlap of the top ten most frequently selected TF triplets for the three different choices for the subset size of TFs that can be probed k. (g+h) Average co-expression within random TF triplets (n = 10,000) and the top 10 triplets chosen most frequently by the different algorithms for the T-cell mediated immunity network (g) and lymphoid leukemia network (h). The co-expression was calculated as the Pearson correlation coefficient on the EpiATLAS RNA-seq data. For each triplet, the co-expression between all three possible TF pairs was taken and then averaged.

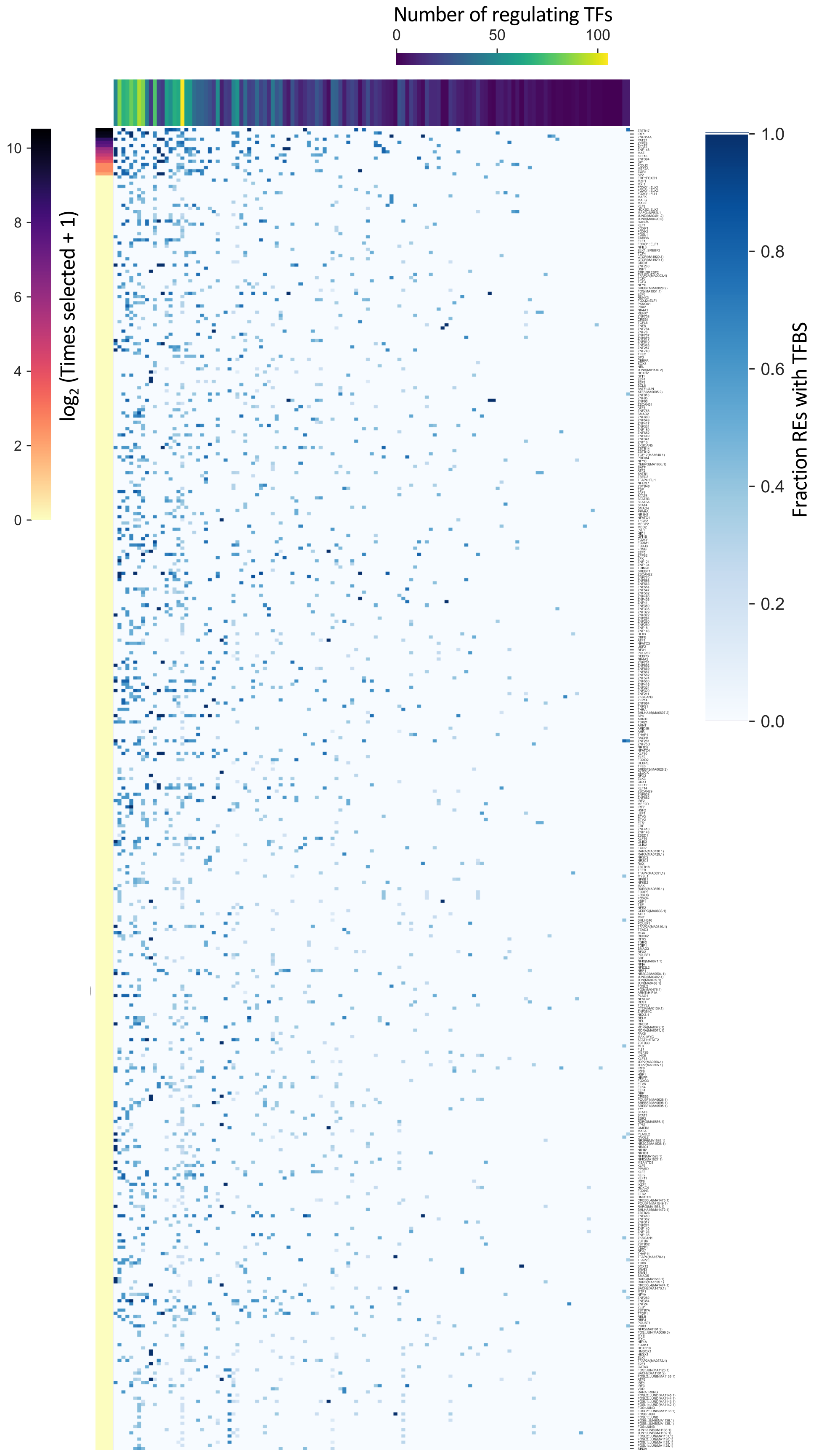

Fig. 7 Visualization of the full network for genes annotated for T-cell mediated immunity. The first row shows the number of transcription factors (TFs) that regulate a gene. The first column is the number of times a TF was part of a TF triplet selected by NampCov (as log_2 with pseudocount).

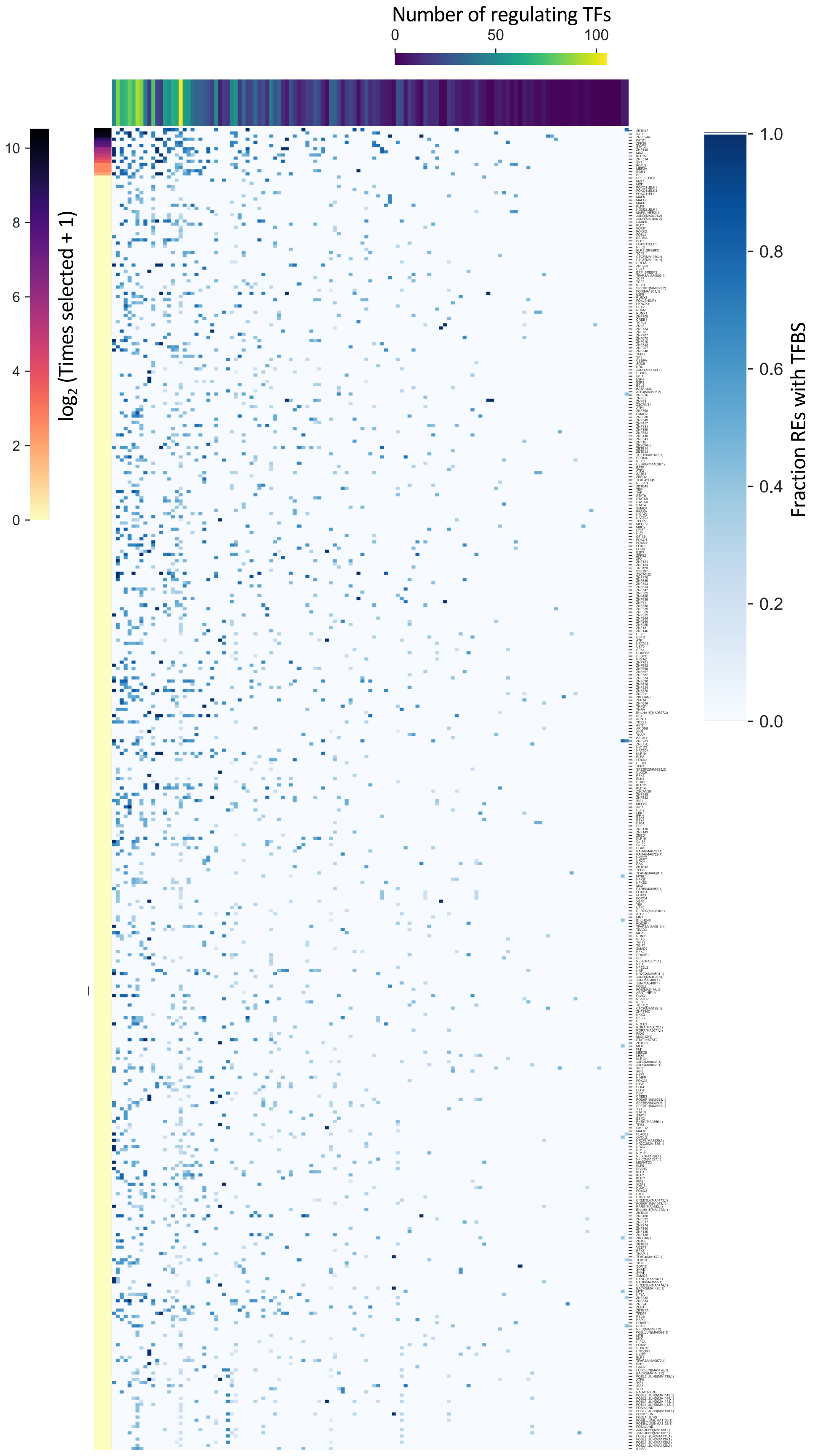

Fig. 8 Visualization of the full network for genes annotated for lymphoid leukemia. The first row shows the number of transcription factors (TFs) that regulate a gene. The first column is the number of times a TF was part of a TF triplet selected by NampCov (as log_2 with pseudocount).

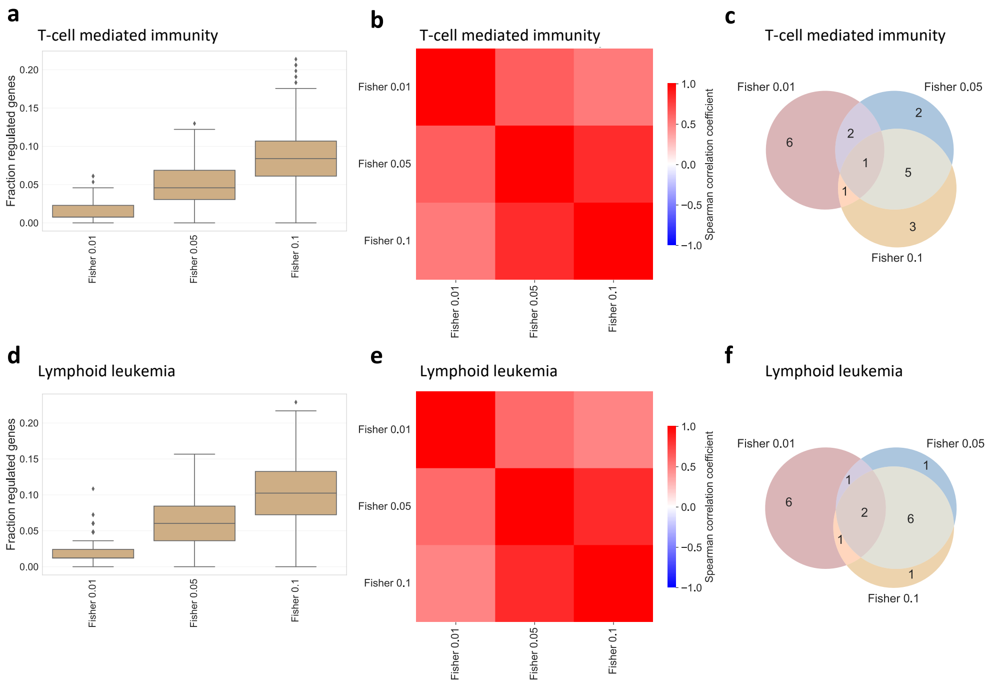

Fig. 9 Network similarity when varying the threshold for network construction. We tested three thresholds for the Fisher's exact test $\{0.01, 0.05, 0.1\}$, which tests whether a transcription factor's (TF) binding sites (TFBS) are enriched in the regulatory elements (REs) of a gene. For all other results in the manuscript we used a cutoff of 0.05. We constructed networks for the three cutoffs and for both gene sets and compared the results from NampCov with $\ell = 3$. (a+d) Fraction of genes covered (≥ 1 non-zero edge) by TFs for varying cutoffs for the T-cell mediated immunity (a) and lymphoid leukemia (d) networks. Boxplots showing center line median, boxlimits inter-quartile range, whiskers up to 1.5x inter-quartile range. (b+e) Spearman correlation coefficient of the fraction of covered genes across TFs between varying networks. (c+f) Overlap of the top ten most frequently selected TFs between varying networks.

4 Algorithm Pseudocodes

Algorithm 1: AMP [3] for f_{max}, f_{sum}

Precompute random distributions $X_1, ..., X_n$ for each *regulator* according to f_{max}, f_{sum}
Input: Random Variables $X_1, ..., X_n$, and parameters k, ℓ
Output: The highest sum achieved by a subset $S \subseteq A$ of ℓ probed variables

```
begin
    S := ∅
    for i ← 1 to k do
        if i ≤ ℓ then
            j̃ := argmax_{j∈[n]\S} (E[X_j])
            Probe X_j̃ and observe realization x_j̃
            S = S ∪ {j̃}
        else
            j̃ := argmax_{j∈[n]\S} ( P[X_j > x^{max}_{S_ℓ}] · E[X_j | X_j > x_{S^ℓ_min}] )
            Probe X_j̃ and observe x_j̃
            S = S ∪ {j̃}
    Sort S according to their realizations in descending order
    return Σ_{j∈[ℓ]} x_{S[j]}
```

Algorithm 2: NAMP [3] for f_{max}, f_{sum}

Precompute random distributions $X_1, ..., X_n$ for each *regulator* according to f_{max}, f_{sum}
Input: Random Variables $X_1, ..., X_n$, and parameters k, ℓ
Output: The highest sum achieved by a subset $S \subseteq A$ of ℓ probed variables

```
begin
    Sort X_i in non-increasing order by E[X_i]
    Probe the first k regulators in this order
    Choose the ℓ regulators among the probed ones with highest realization
    return the sum of those regulators
```

Algorithm 3: GREEDY/OFF for f_{cov} [4]

Input: Sets A, B with weights $w_{a,b} \geq 0$ for every $a \in A, b \in B$ and parameter ℓ
Output: Highest coverage sum (approximated) of any subset $S \subseteq A$ of cardinality $\leq k$

```
begin
    S := ∅
    w_b^max = 0 for all b ∈ B
    for i ← 1 to ℓ do
        ã := arg max_{a∈A\S} (∑_{b∈B} max{0, w_{a,b} − w_b^max})
        w_b^max = max{0, w_{ã,b} − w_b^max}
        S = S ∪ {ã}
    return ∑_{b∈B} w_b^max
```

Algorithm 4: AMPCOV for f_{cov} [3]

Input: BIPARTITEREGULATORPROBING instance with random variables $D_{a,b}$, and parameters k, ℓ
Output: The highest coverage sum achieved by a subset $S \subseteq A$ of ℓ *probed regulators* approximated by GREEDY

```
begin
    S := ∅
    w_b^max = 0 for all b ∈ B
    for i ← 1 to k do
        if i ≤ ℓ then
            ã := argmax_{a∈A\S} (∑_{b∈B} ℙ[D_{a,b} > w_b^max] · 𝔼[D_{a,b} | D_{a,b} > w_b^max])
            Probe ã and observe realizations w_{ã,b} for all b ∈ B
            Update w_b^max for all b ∈ B accordingly
            S = S ∪ {ã}
        else
            // 𝔼[a] refers to setting all incident edges of a to
               their expected values
            ã := arg max_{a∈A\S} (GREEDY (S ∪ {𝔼[a]}))
            Probe ã and observe realizations w_{ã,b} for all b ∈ B
            S = S ∪ {ã}
    return GREEDY(S)
```

Algorithm 5: NampCov for f_{cov} [3]

Input: BipartiteRegulatorProbing instance with random variables $D_{a,b}$, and parameters k, ℓ

Output: The highest coverage sum achieved by a subset $S \subseteq A$ of ℓ *probed regulators* approximated by Greedy

```
begin
    S := ∅
    w_b^max = 0 for all b ∈ B
    for i ← 1 to k do
        if i ≤ ℓ then
            ã := arg max_{a∈A\S} ( Σ_{b∈B} P[D_{a,b} > w_b^max] · E[D_{a,b} | D_{a,b} > w_b^max] )
            Simulate a Probe ã by "observing" w_{ã,b} = E[D_{ã,b}] for all b ∈ B
            Update w_b^max for all b ∈ B accordingly
            S = S ∪ {ã}
        else
            ã := arg max_{a∈A\S} ( Σ_{b∈B} E[D_{a,b}] )
            S = S ∪ {ã}
    return Greedy(S)
```

$$
\begin{aligned}
\text{maximize} \quad & \sum_{a\in A}\sum_{b\in B} y_{a,b}\cdot w_{a,b} & \\
\text{subject to} \quad & \sum_{a\in A} y_{a,b} <= 1, & \forall b\in B\\
& y_{a,b} <= x_a, & \forall a\in A, b\in B\\
& \sum_{a\in A} x_a <= \ell & \\
& x_a \in 0,1, y_{a,b}\in[0,1] & \forall a\in A, b\in B
\end{aligned}
$$

Fig. 10 Integer program (IP) for weighted MaximumCoverage. Variables x_a represent the appearance of a *regulator* $a \in A$ in the final set S. Variables $y_{a,b}$ model the appearance of an edge in the *final covering* of S: if $\tilde{a} = \arg\max_{a\in S} w_{a,b}$ for $b \in B$, then $y_{\tilde{a},b} > 0\,(= 1)$. Relaxing $x \in [0,1]$ yields an upper-bound used as Opt for f_{cov}.

References

[1] Fu, H., Li, J., Xu, P.: A PTAS for a Class of Stochastic Dynamic Programs. In: Chatzigiannakis, I., Kaklamanis, C., Marx, D., Sannella, D. (eds.) 45th International Colloquium on Automata, Languages, and Programming (ICALP 2018). Leibniz International Proceedings in Informatics (LIPIcs), vol. 107, pp. 56–15614 (2018). https://doi.org/10.4230/LIPIcs.ICALP.2018.56 . https://drops.dagstuhl.de/entities/document/10.4230/LIPIcs.ICALP.2018.56

[2] Segev, D., Singla, S.: Efficient approximation schemes for stochastic probing and prophet problems. In: Proceedings of the 22nd ACM Conference on Economics and Computation. EC '21, pp. 793–794. Association for Computing Machinery, New York, NY, USA (2021). https://doi.org/10.1145/3465456.3467614 . https://doi.org/10.1145/3465456.3467614

[3] Asadpour, A., Nazerzadeh, H.: Maximizing stochastic monotone submodular functions. Manag. Sci. **62**(8), 2374–2391 (2016) https://doi.org/10.1287/MNSC.2015.2254

[4] Nemhauser, G., Wolsey, L., Fisher, M.: An analysis of approximations for maximizing submodular set functions—i. Mathematical Programming **14**, 265–294 (1978) https://doi.org/10.1007/BF01588971
